# A Solid-State Thin-Film Ag/AgCl Reference Electrode Coated with Graphene Oxide and Its Use in a pH Sensor

**DOI:** 10.3390/s150306469

**Published:** 2015-03-17

**Authors:** Tae Yong Kim, Sung A Hong, Sung Yang

**Affiliations:** 1 Department of Medical System Engineering, Gwangju Institute of Science and Technology (GIST), Gwangju 500-712, Korea; E-Mails: tykim9544@gist.ac.kr (T.Y.K.); griefskysea@gist.ac.kr (S.A.H.); 2 School of Mechatronics, Gwangju Institute of Science and Technology (GIST), Gwangju 500-712, Korea

**Keywords:** Ag/AgCl, reference electrode, pH sensor, graphene oxide, iridium oxide pH working electrode, potentiometer

## Abstract

In this study, we describe a novel solid-state thin-film Ag/AgCl reference electrode (SSRE) that was coated with a protective layer of graphene oxide (GO). This layer was prepared by drop casting a solution of GO on the Ag/AgCl thin film. The potential differences exhibited by the SSRE were less than 2 mV for 26 days. The cyclic voltammograms of the SSRE were almost similar to those of a commercial reference electrode, while the diffusion coefficient of Fe(CN)_6_^3−^ as calculated from the cathodic peaks of the SSRE was 6.48 × 10^−6^ cm^2^/s. The SSRE was used in conjunction with a laboratory-made working electrode to determine its suitability for practical use. The average pH sensitivity of this combined sensor was 58.5 mV/pH in the acid-to-base direction; the correlation coefficient was greater than 0.99. In addition, an integrated pH sensor that included the SSRE was packaged in a secure digital (SD) card and tested. The average sensitivity of the chip was 56.8 mV/pH, with the correlation coefficient being greater than 0.99. In addition, a pH sensing test was also performed by using a laboratory-made potentiometer, which showed a sensitivity of 55.4 mV/pH, with the correlation coefficient being greater than 0.99.

## 1. Introduction

Electrochemical sensors fabricated using microfabrication techniques have been receiving much attention because they are generally simple to make, portable, and cost effective [1]. The reference and working electrodes are crucial components of any electrochemical sensor system. Conventionally, in the sensors used for pH testing and for oxidation-reduction potential sensing, a liquid-junction Ag/AgCl reference electrode is combined with the working electrode in a glass body, as this results in high potential stability. A report on the features and characterization of several types of pH sensor was presented in [2]. Of the various types, the solid-state reference electrode (SSRE) has been known to reduce the problems associated with conventional reference electrodes relating to mass-production, maintenance, contamination by the internal solution, and miniaturization [3]. SSREs have emerged as alternatives to the traditionally used electrodes, owing to the simplicity and ease of fabrication of the former. Thin-film Ag/AgCl reference electrodes that can be used in microfabricated electrochemical sensors have been developed [4,5,6,7,8,9,10,11]. There are several methods of fabricating electrodes, such as electrochemical deposition [5,12], screen-printing [9,13,14], and sputter coating [4,6]. It is known that the electrodes fabricated by electrochemical deposition consist of amorphous films with varying levels of hydration and relatively high internal microporosities [15]. The screen-printing technique, which has been used to synthesize a few types of electrodes, including disposable ones, is a useful method owing to its simplicity and low-cost nature [16]. However, the screen-printing technique is limited in that only a few metals can be deposited on the substrate. On the other hand, the electrodes fabricated by sputter coating are known to be chemically stable [17]. Sputter coating is thus widely used for electrode fabrication [18,19,20]. In addition, this method is also compatible with the microfabrication processes involved in the fabrication of micro total analysis systems (μTAS). Regardless of the fabrication methods used, the potential of the reference electrode should remain stable during electrochemical sensing. However, when the electrode is dipped in the test solution, the AgCl present on the thin Ag film gradually dissolves at high chloride-ion concentrations [5,21,22]. This can lead to the potential of the reference electrode becoming unstable and thus cause the working electrode to give erroneous readings. Thus, protective layers such as those made of Nafion and various polymers are deposited on the thin-film Ag/AgCl electrode to prevent the dissolution of AgCl [4,8,10,11,17,21,23,24]. Out of all these coating materials, Nafion is the most frequently used one. However, this material has been shown to have poor reproducibility when used with SSREs [12]. It is also expensive to manufacture, given the complexity of its synthesis process [25].

In this study, we employ graphene oxide (GO) as an alternative protective layer to coat a solid-state thin-film Ag/AgCl reference electrode. This layer is formed simply by drop casting a GO dispersion and drying the resultant coating. When GO dispersed in water is coated and dried on a substrate, it forms laminates with interlocked layered structures that exhibit good mechanical strength [26]. Electrochemical investigations are performed to characterize the performance of the SSRE coated with GO. Further, a pH sensor consisting of the proposed SSRE and a laboratory-made pH working electrode is prepared and used for a pH sensing test. In addition, an integrated pH sensor is fabricated on a chip and packaged in a secure digital (SD) card, which is a portable data storage device. We manufactured a laboratory-made potentiometer based on the integrated pH sensor in order to verify the applicability of the sensing system for practical use; the sensor and the potentiometer showed satisfactory pH sensing performance, as the results showed that the performance of the sensor is comparable to that of a commercial one.

## 2. Experimental Section 

### 2.1. Fabrication of the SSRE

**Figure 1:**
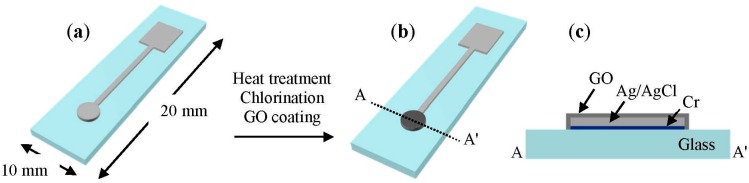
Schematic of the SSRE fabrication process. The sensing part of the electrode is 2 mm in diameter. (**a**) Deposition of Cr and Ag by a sputter process; (**b**) SSRE coated with graphene oxide; (**c**) Cross-sectional view of the electrode.

The process for fabricating the SSRE is described in Figure 1. A 30-nm-thick adhesive layer of Cr (Thifine, Incheon, Korea) and then a 1000-nm-thick layer of Ag (Thifine, Incheon, Korea) were deposited on a glass substrate using radio frequency sputtering and direct-current sputtering (A-Tech System, Incheon, Korea), respectively. A heat treatment was performed after the sputter-coating step during the fabrication process to improve the mechanical strength of the Ag thin film, because untreated thin films of Ag or Ag/AgCl often get stripped off from the substrate during fabrication or experimentation. After testing different temperatures, the heat treatment temperature was set to 320 °C. The treatment time was 30 min, and the treatment was performed in a flow of nitrogen gas (~0.2 MPa) at ambient pressure in an electric furnace (LEF-112S, Labtech, Namyangju, Korea). Then, the electrode was allowed to cool to room temperature. As the next step, a drop of 50 mM ferric chloride (FeCl_3_) (5021-4400, Dae Jung Chemicals & Metals, Siheung, Korea) solution was placed on the Ag surface and left there for 50 s to chlorinate the Ag layer and grow AgCl layer on it. The chlorinated electrodes were stored in a saturated AgCl solution overnight and then washed in running deionized (DI) (Milli-Q Academy, Millipore S.A.S., Molsheim, France) water and dried. GO (Graphene Supermarket, Calverton, NY, USA) was purchased in the form of dried flakes and dispersed in DI water to reach a concentration of 1.375 g/L through ultrasonication for 1 h at room temperature. Next, 20 μL of this GO suspension was carefully placed on the Ag/AgCl thin film and dried on a hotplate heated to 40 °C. A sensing area with a diameter of 2 mm, which was coated with GO, was formed on the glass substrate. 

**Figure 2:**
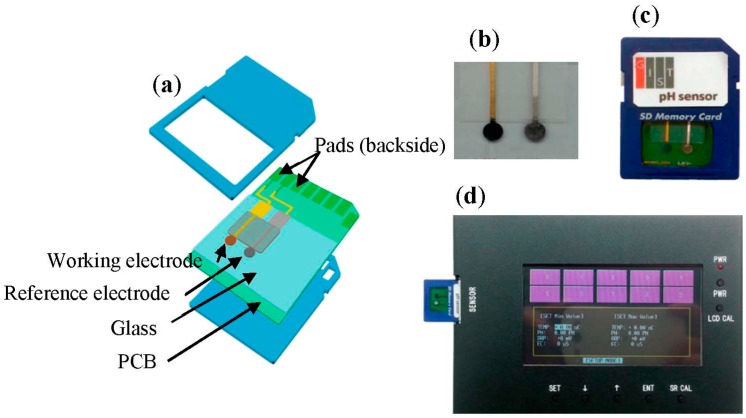
The SSRE coated with GO and the pH working electrode were built on a square-shaped glass substrate. (**a**) Schematic showing the individual parts of the sensor; (**b**) Photograph of the actual sensor fabricated on a glass substrate; (**c**) Photograph of the sensor packaged in an SD card; (**d**) Laboratory-made potentiometer that was used in the integrated pH sensor.

Finally, a pH sensor comprising the SSRE and a pH working electrode were fabricated (Figure 2); the fabrication details are mentioned in Section 2.4. An Orion Ag/AgCl reference electrode (ORE) (900100, Thermo Scientific, Beverly, MA, USA) was also used for comparison. 

### 2.2. Observation of Surface Morphology of SSRE

The changes in the surface morphology of the SSRE resulting from the following fabrication processes were investigated using scanning electron microscopy (SEM) (Quanta 200F, FEI, Hillsboro, OR, USA): before and after the heat treatment, after chlorination with ferric chloride solution, and after being stored in a saturated AgCl solution. The surface features observed after each step are described, and the corresponding images are shown in Section 3.1. The surface of the heat-treated electrode was also observed with an atomic force microscopy (AFM) system (XE-100, Park Systems, Suwon, Korea) before and after it was coated with GO. An area of 30 × 30 μm was observed during imaging. 

### 2.3. Open-Circuit Potential and Cyclic Voltammetry

An electrochemical workstation (CHI760D, CH Instruments, Austin, TX, USA) was used to measure the open-circuit potential of the electrodes and to perform cyclic voltammetry (CV). The SSRE and ORE were connected to the working and reference electrical lines, respectively, of the electrochemical workstation. During the CV measurements, the SSRE or the ORE was used as the reference electrode, a thin-film gold electrode 2 mm in width and 3 mm in length was employed as the working electrode, and a commercially obtained Pt wire (CH Instruments, Austin, TX, USA) was used as the counter electrode. The SSRE and ORE were used alternatively as the reference electrode so that their performances could be compared. The CV measurements were performed using a redox couple consisting of 1 mM potassium ferricyanide (K_3_Fe(CN)_6_) (702587, Sigma-Aldrich, St. Louis, MO, USA) in 1 M potassium chloride (KCl) (DC Chemical, Seoul, Korea) at scan rates of 25, 50, 100, 150, and 200 mV/s. To prepare the pH solutions, a buffer solution containing 5 mM potassium hydrogen phthalate (179922, Sigma-Aldrich, St. Louis, MO, USA), 5 mM potassium dihydrogen phosphate (P5655, Sigma-Aldrich, St. Louis, MO, USA), 5 mM tris(hydroxymethyl)-aminomethane (252859, Sigma-Aldrich, St. Louis, MO, USA), 2.5 mM sodium tetraborate decahydrate (S9640, Sigma-Aldrich, St. Louis, MO, USA), and 100 mM NaCl (71376, Sigma-Aldrich, St. Louis, MO, USA) was used. The pH levels of the solutions were adjusted by adding 1 M HCl (320331, Sigma-Aldrich, St. Louis, MO, USA) or 1 M NaOH (221465, Sigma-Aldrich, St. Louis, MO, USA) and were measured with a commercial pH meter (ORION 5 STAR, Thermo Electron, Madison, WI, USA). 

### 2.4. Fabrication of Laboratory-Made pH Sensor and Potentiometer

A laboratory-made pH sensor, which consisted of the SSRE and an iridium oxide pH working electrode [27], was prepared. To fabricate the working electrode, the solution for the electrodeposition of the working electrode was prepared as follows: 0.15 g of iridium (IV) chloride hydrate (IrCl_4_·H_2_O) (51996, Sigma-Aldrich, St. Louis, MO, USA) was dissolved in DI water under magnetic stirring for 30 min; this was followed by the addition of 1 mL of 30% hydrogen peroxide (H_2_O_2_) (H1009, Sigma-Aldrich, St. Louis, MO, USA) to the solution under stirring for 30 min. Next, 0.5 g of oxalic acid (C_2_H_2_O_4_) (247537, Sigma-Aldrich, St. Louis, MO, USA) was added, and the solution was stirred for 30 min. The solution pH was adjusted to ~9.7 by adding lithium carbonate (Li_2_CO_3_) (255823, Sigma-Aldrich, St. Louis, MO, USA). Electrodeposition onto a Au sensing area was then carried out using a current of 0.01 mA for 20 min at room temperature; a Au electrode 2 mm in diameter prepared by sputtering was used as the substrate. The electrodeposited iridium oxide electrode was subsequently heat treated at 400 °C for 30 min in a supply of nitrogen gas (~0.2 MPa) at ambient pressure in the electric furnace. 

Two types of pH sensors, namely, a combined sensor and an integrated sensor, were prepared to verify the suitability of the SSRE for pH sensing. The working and reference electrodes were fabricated separately and combined together, which was comprising the pH sensor. On the other hand, in the case of the integrated pH sensor, both the electrodes were fabricated on the same chip in parallel using a serial microfabrication process; the working electrode was fabricated first and then the SSRE. This integrated pH sensor was then packaged on the printed circuit board (PCB) of an SD card (512M, Toshiba, Tokyo, Japan) after removing the memory chip (Figure 2). Each electrode of the sensor was connected to the contact pad of the SD card with gold wires (0.2 mm in diameter). Then, this pH sensor was inserted into an SD card connector of a potentiometer to make the potential measurements which was manufactured in the laboratory and used with the integrated sensor to make pH measurements (Figure 2d). 

## 3. Results and Discussion

### 3.1. Surface Morphology and Chemical Analysis of SSRE

**Figure 3:**
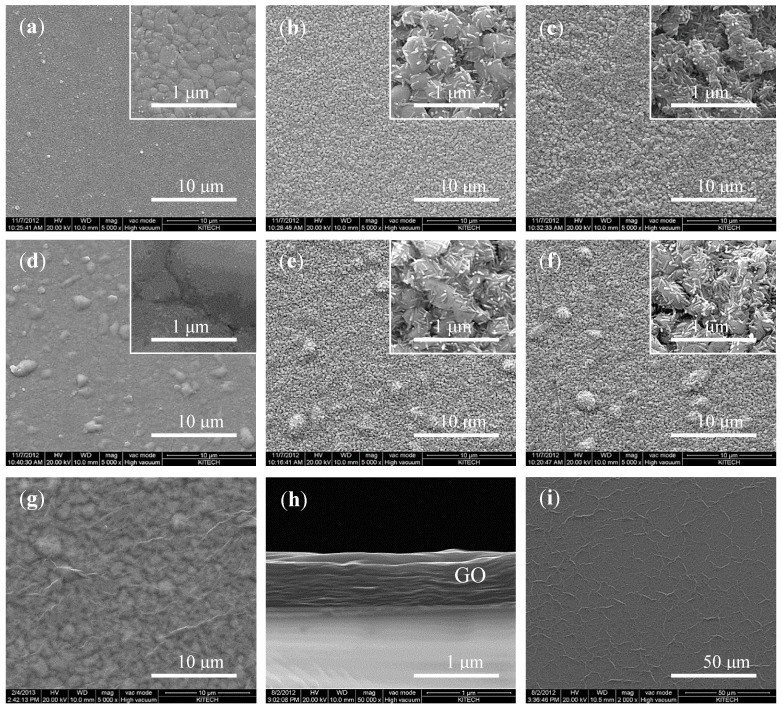
Changes in the surface morphology of the electrode as observed using SEM: (**a**–**c**) non-heat-treated electrodes and (**d**–**f**) electrodes heat-treated at 320 °C. (The magnification of the images in (**a**–**g**) is × 5 K, that for images in the insets of (**a**–**f**) and (**h**) is × 50 K, and that for the image in (**i**) is × 2 K. Images of the Ag and Ag/AgCl thin films after the following steps are shown: (**a**) before the heat treatment, (**d**) after the heat treatment, (**b**,**e**) after chlorination with 50 mM FeCl_3_, and (**c**,**f**) after overnight storage in a saturated AgCl solution. (**g**) GO layer on a ready-to-use electrode. (**h**) and (**i**) show cross-sectional and top views of the pristine GO layer, respectively.

The surface of the as-deposited Ag thin film, which was produced *via* a sputtering process, was primarily flat and lustrous. The surfaces of the Ag and Ag/AgCl thin films were observed by SEM to investigate the changes in their surface morphologies. The surface morphologies were observed at each step in the fabrication process (Figure 3). The SEM images of the heat-treated electrode showed that larger and more tightly packed Ag granules facing the sides were present on its surface (Figure 3d). After the chlorination process, small rod-shaped structures were observed on both electrodes (Figure 3b,e). In contrast to a report that a greater number of grains grow on the AgCl layer after the electrode has been stored overnight in a saturated AgCl solution [28], we did not observe significant differences in the images of the electrode before and after it was placed overnight in a saturated AgCl solution (Figure 3e,f). We assume that this is attributable to the fact that the method used to deposit the Ag/AgCl thin film in the report (electroplating) was different from the one used in the present study (sputter coating). 

**Figure 4:**
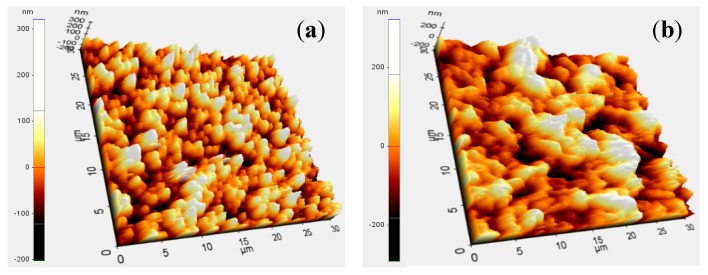
AFM images of the SSRE showing its surface morphology. Images (**a**) and (**b**) show the morphology before and after the electrode was coated with GO, respectively.

The GO layers formed in four trials had an average thickness of 756 (±23.1) nm (Figure 3h), which was comparable to that in the previous report [11]; however, it was not clear how poor the reproducibility of the Nafion layer was. The average roughness (R_a_) values of the electrode surface before and after it was coated with the GO layer, as determined from AFM images, were 50.2 and 73.4 nm, respectively (Figure 4). The finer peaks and valleys were formed by the small granules of Ag/AgCl while the bigger bumps were probably due to the wrinkle-like structures of the GO layer that was coated on the Ag/AgCl thin film. 

**Figure 5:**
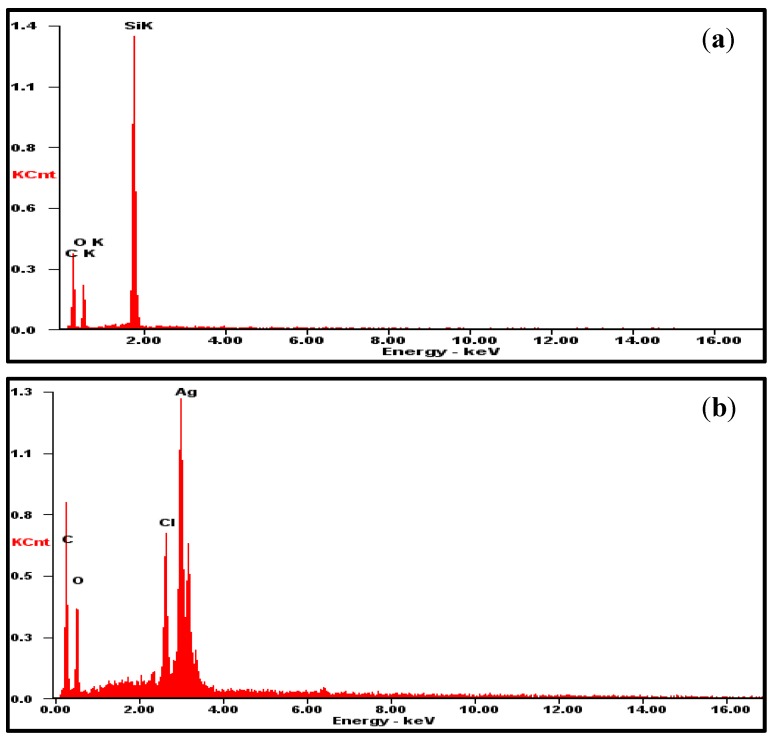
EDS analysis of the electrode surface. (**a**) GO layer formed on a silicon wafer (shown for comparison); (**b**) GO layer on a thin film of Ag/AgCl.

It can be surmised that the R_a_ value of the surface shown in Figure 4b is greater than that of the surface shown in Figure 4a, because the wrinkle-like structures, which were observed on a pristine GO layer (Figure 3i), still existed on the surface of the protective layer deposited on the Ag/AgCl thin film. In addition, a chemical analysis of the GO layer on the SSRE was performed using energy dispersive spectrophotometry (EDS). The elements Ag, Cl, C, and O were detected, as expected (Figure 5). From the results, it can be surmised that a GO layer was indeed formed on the surface of the Ag/AgCl thin film.

### 3.2. Effect of pH on the SSRE and the Long-Term Stability of the SSRE

**Figure 6:**
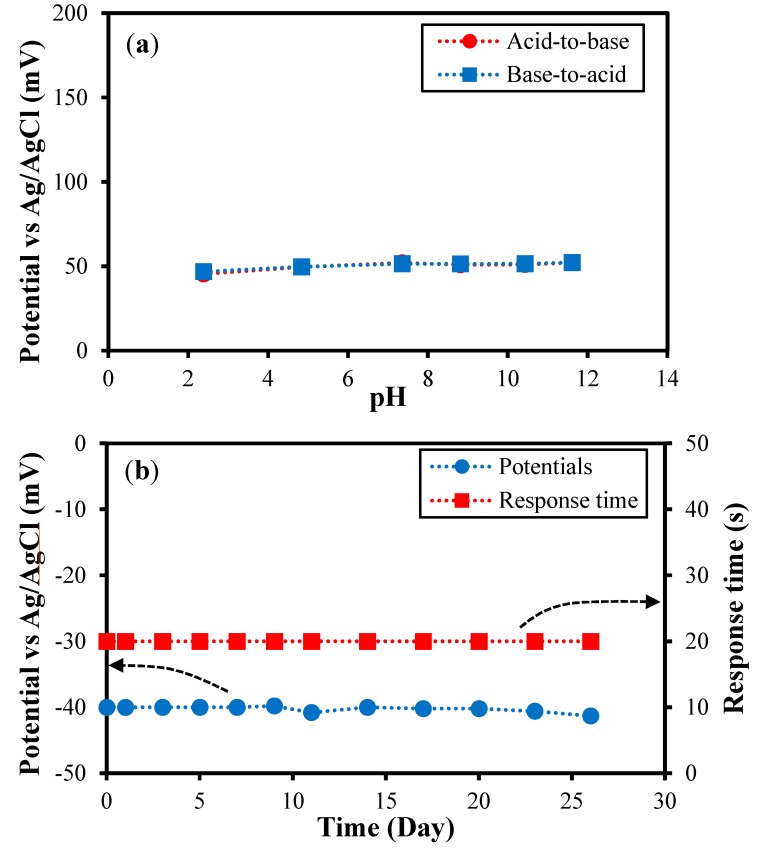
Effect of pH on the SSRE and the long-term stability of the SSRE. (**a**) Stability of the SSRE at pH levels ranging from 2.38 to 11.61 in the acid-to-base direction and *vice versa*. The potentials were measured using the ORE as the reference electrode; (**b**) Potentials and response times of the SSRE were measured in a 3 M KCl solution at intervals of 2 or 3 days over 26 days.

The potential of the SSRE was evaluated in solutions with pH values ranging from 2.38 to 11.61, both in the acid-to-base direction and *vice versa* (Figure 6a), to determine whether the potential was affected by the pH change. The electrode was dipped in the solutions for 60 s. The potentials, which were measured versus the ORE as the reference electrode, remained at approximately 50 mV, varying by less 5 mV over the pH range. This indicated that the potential of the SSRE was almost independent of the given pH range.

The long-term stability of a reference electrode is important with respect to its electrochemical sensor performance. The potential and response time of the SSRE were measured at intervals of 2 or 3 days as functions of the time over a period of 26 days using a 3 M KCl solution (Figure 6b). The potential of the SSRE varied by less than 2 mV, indicating that the potential remained quite stable over the test period. Moreover, the potential became stable within 20 s after the electrode had been dipped into the solutions during each of the measurements. According to a previous report [29], a number of capillary channels with a spacing of 0.7 to 1.1 nm, depending on the relative humidity per unit area, are formed in the GO layer, and water molecules from the test solution can diffuse through these channels. In addition, because GO has negative ions that are oxygen-containing functional groups on its basal plane and edges, the GO layer functions as a protective layer, hindering the diffusion of the released chloride ions from the electrode surface, which is negatively charged. 

### 3.3. Cyclic Voltammetry Measurements

**Figure 7:**
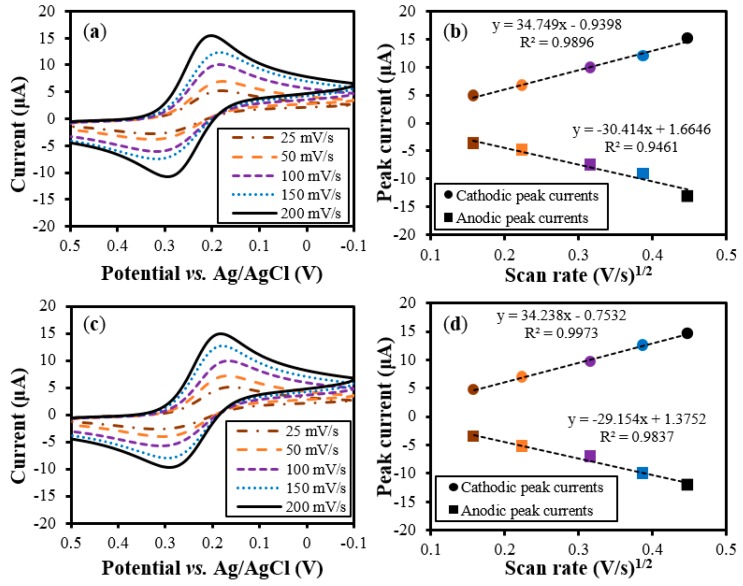
Comparison of the CV curves of the ORE and SSRE for scan rates of 25, 50, 100, 150 and 200 mV/s. (**a**) and (**b**) are the CV curves for the ORE (plotted for comparison), and (**c**) and (**d**) are the curves for the SSRE. The solid circles (●) and boxes (■) in (**b**) and (**d**) stand for the cathodic and anodic peak currents, respectively. (The measurements were made three times. The error bars for the values were not shown as the difference in the currents was smaller than 0.5 μA.)

CV measurements were performed at the scan rates of 25, 50, 100, 150, and 200 mV/s using the ORE and SSRE in a redox couple consisting of 1 mM K_3_Fe(CN)_6_ in 1 M KCl to explore the potential of using the SSRE as a reference electrode in real-world electrochemical applications (Figure 7). Cyclic voltammograms were obtained using the ORE and the SSRE; these are shown in Figure 7a and Figure 7c, respectively. The peak currents increased with an increase in the scan rates; this was true for both the electrodes. In addition, it was found that the peak potentials changed slightly with the increase in the scan rate. This indicates that, at higher scan rates, the redox reaction can be considered as a quasi-reversible one. The anodic and cathodic peak currents for the ORE and SSRE were plotted with respect to the square root of the scan rates in Figure 7b,d, respectively, to calculate and compare the diffusion coefficients of the charged substance, ferricyanide (Fe(CN)_6_^3−^), in the test solution. The peak currents were linearly proportional to the square root of the scan rate. As a result, the peak currents for the ORE and SSRE were comparable to each other, exhibiting variations smaller than 0.5 μA at each measurement. The diffusion coefficient of ferricyanide as calculated from the cathodic peaks was 6.48 × 10^−6^ cm^2^/s in the case of the SSRE, which is slightly lower than the theoretical value of 7.6 × 10^−6^ cm^2^/s [30]; this was probably due to the quasi-reversibility of the redox reaction at higher scan rates. In particular, the diffusion coefficient in the case of the SSRE was close to that for the ORE (6.46 × 10^−6^ cm^2^/s), meaning that the performance of the SSRE was comparable to that of the commercial reference electrode. 

### 3.4. Performances of the Laboratory-Made pH Sensor with the SSRE

**Figure 8:**
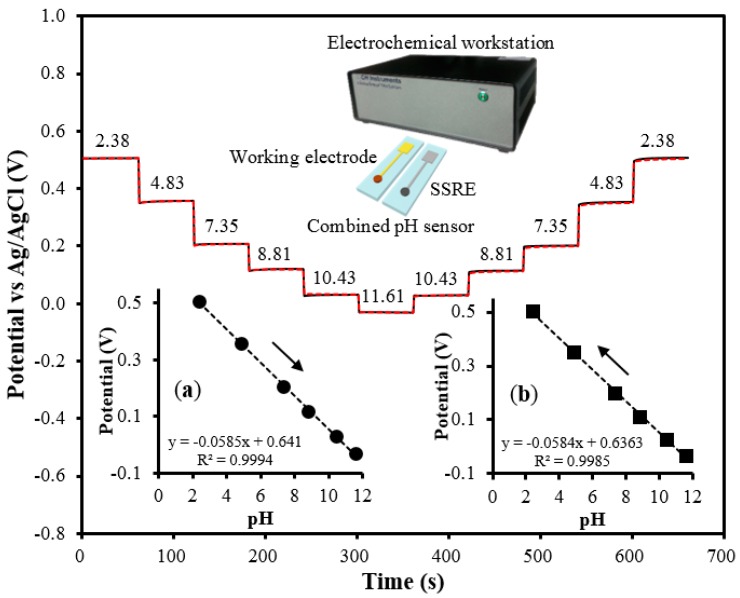
The combined pH sensor and the electrochemical workstation were used for measuring the potentials. The potentials measured with the pH sensor are represented by the straight (―) and dotted (---) lines. The numbers in the graph indicate the pH. (**a**) and (**b**) show the proportional relation between the pH and the potentials in the acid-to-base direction and *vice versa*, respectively (the measurements were made three times, and the arrows indicate the direction for pH sensing).

Two combined pH sensors were used for pH sensing (Figure 8). Each of the two SSREs was alternatively combined with the same working electrode comprising the pH sensor to evaluate the performance of the SSREs. These combined sensors were then employed to measure the potentials of solutions with pH values ranging from 2.38 to 11.61; the tests were performed both in the acid-to-base direction and *vice versa* and the potentials were measured three times for each solution. 

The potentials from the two sensors, which exhibited almost complete overlapping, showed differences of less than 5 mV, indicating good reproducibility. The sensitivity of the sensors was also investigated, as shown in Figure 8a,b. The average pH sensitivities were 58.5 and 58.4 mV/pH in the acid-to-base and the base-to-acid directions, respectively; this meant that almost one electron per H^+^ ion was transferred during the redox reaction, resulting in a near-Nernstian response. Further, these values were comparable to the theoretical sensitivity of 59 mV/pH. Further, the correlation coefficients in both the cases were greater than 0.99. Thus, it was concluded that these pH sensors showed promising performances, which were mostly attributable to the properties of the SSRE, implying that it was suited for use as a reference electrode. 

**Figure 9:**
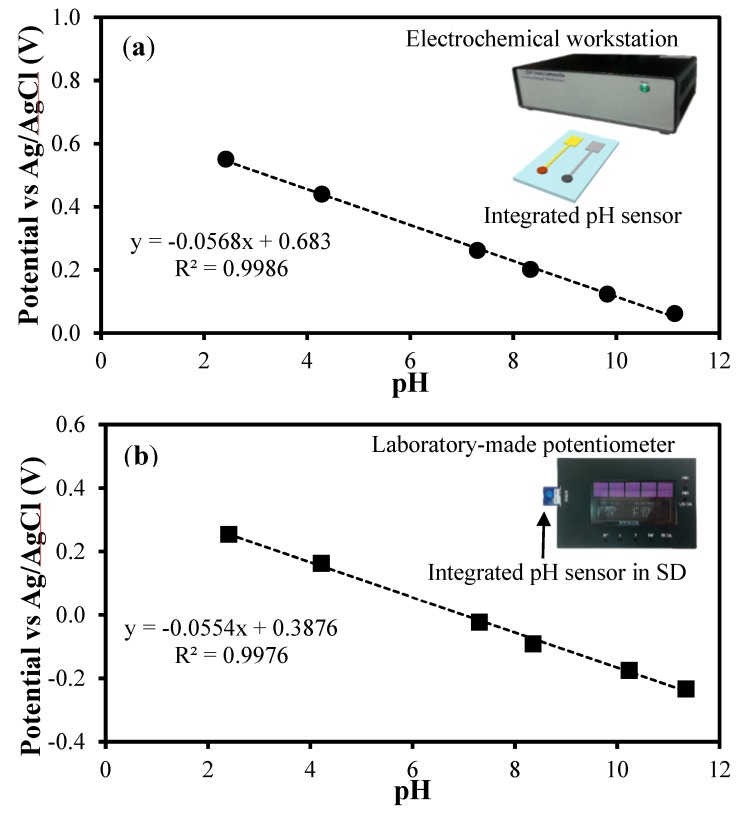
The integrated pH sensors were evaluated in various solutions of different pH values. (**a**) The integrated pH sensors with an electrochemical workstation, and (**b**) the integrated pH sensors packaged in an SD card with the laboratory-made potentiometer were used for potential measurements. Each inset indicates the experimental setup used for the test. (The error bars were not shown as the difference in the potentials was smaller than 10 mV for both (**a**) and (**b**).)

On the other hand, two integrated pH sensors were packaged in SD cards and were tested (Figure 9a). For the potential measurements, solutions with pH values ranging from 2.45 to 10.0 were used. The average pH sensitivity was 56.8 mV/pH, with the correlation coefficient being greater than 0.99, resulting in a near-Nernstian response. In addition, the potential difference between the two individual sensors was less than 10 mV, indicating high reproducibility. 

Finally, two integrated pH sensors were evaluated using the laboratory-made potentiometer and solutions with pH values ranging from 2.40 to 11.34. The average pH sensitivity was 55.4 mV/pH; this value was comparable to the theoretical value, with the correlation coefficient being greater than 0.99 (Figure 9b). The potential difference was less than 10 mV between two measurements, indicating good reproducibility. Although the sensitivity value was slightly smaller than 56.2 mV/pH, which was value obtained with the same pH sensor when the CHI electrochemical workstation was used for the potential measurement, this pH sensing system consisting of the pH sensor and potentiometer was thought to be suitable for practical use.

## 4. Conclusions

In this study, we fabricated a novel SSRE coated with a layer of GO. The results of CV measurements and pH sensing tests, performed with laboratory-made sensors, suggested that the proposed SSRE could be used as a reference electrode in electrochemical applications. Above all, the promising performance of the pH sensors based on the electrode could primarily be attributed to the properties of the SSRE. Furthermore, we expect that this electrode will be utilized in miniaturized electrochemical sensors as well, because the processes for fabricating the SSRE and the working electrode are compatible with conventional semiconductor production processes. 

We developed a laboratory-made potentiometer for the verification of the performance of the pH sensor. Though the experimental results confirmed that the instrument can be used with the pH sensor, it is needed to improve both the sensor and instrument for better performance. We hope to realize a device with multisensing capability to sense not only the pH level but also the oxidation-reduction potential, electrical conductivity, and temperature. We are currently trying to improve the instrument and multisensor so that they can be used in real-world applications in the near future.
